# Supplementary material for: Expanding the Clinical and Genetic Spectrum of RAB28-Related Cone-Rod Dystrophy: Pathogenicity of Novel Variants in Italian Families
Source: Int J Mol Sci. 2020 Dec 31;22(1):381. doi: 10.3390/ijms22010381 (PMC7795990; doi:10.3390/ijms22010381)
Supplement: Supplementary file 1 [file ijms-22-00381-s001.zip › Supplementary_files/ijms-1051407 sm.pdf]

International Journal of *Molecular Sciences*

Supplementary Materials

Expanding the Clinical and Genetic Spectrum of *RAB28*-related Cone-Rod Dystrophy: Pathogenicity of Novel Variants in Italian Families

Giancarlo Iarossi[1,*], Valerio Marino[2,*], Paolo Enrico Maltese[3], Leonardo Colombo[4], Fabiana D'Esposito[5,6,7], Elena Manara[6], Kristjana Dhuli[6], Antonio Mattia Modarelli[4], Gilda Cennamo[8], Adriano Magli[9], Daniele Dell'Orco[2,], Matteo Bertelli[3,6]**

Video V1. Three-dimensional structure of GTP-bound WT Rab-28, protein structure is represented as an orange cartoon, Mg^{2+}-ion is shown as an orange sphere, GTP is displayed as magenta sticks, the molecular surface of each element is shown in transparency and colored accordingly.

Video V2. Three-dimensional structure of GTP-bound p.(Arg137*) Rab-28, protein structure is represented as a teal cartoon, Mg^{2+}-ion is shown as a teal sphere, GTP is displayed as magenta sticks, the molecular surface of each element is shown in transparency and colored accordingly. The GTP molecule is merely indicative, as both truncated forms would not allow the correct folding of the binding pocket.

Video V3. Three-dimensional structure of GTP-bound p.(Trp107*) Rab-28, protein structure is represented as a light blue cartoon, Mg^{2+}-ion is shown as a light blue sphere, GTP is displayed as magenta sticks, the molecular surface of each element is shown in transparency and colored accordingly. The GTP molecule is merely indicative, as both truncated forms would not allow the correct folding of the binding pocket.

Publisher's Note: MDPI stays neutral with regard to jurisdictional claims in published maps and institutional affiliations.

© 2020 by the authors. Submitted for possible open access publication under the terms and conditions of the Creative Commons Attribution (CC BY) license (http://creativecommons.org/licenses/by/4.0/).
